# A Prospective Study: Current Problems in Radiotherapy for Nasopharyngeal Carcinoma in Yogyakarta, Indonesia

**DOI:** 10.1371/journal.pone.0085959

**Published:** 2014-01-23

**Authors:** Sharon D. Stoker, Maarten A. Wildeman, Renske Fles, Sagung R. Indrasari, Camelia Herdini, Pieter L. Wildeman, Judi N. A. van Diessen, Maesadji Tjokronagoro, I. Bing Tan

**Affiliations:** 1 Department of head and neck oncology and Surgery, The Netherlands Cancer Institute, Amsterdam, The Netherlands; 2 Department of otorhinolaryngology, Academic Medical Centre, Amsterdam, the Netherlands; 3 Department of otorhinolaryngology, Dr Sardjito General Hospital/Faculty of Medicine Universitas Gadjah Mada, Yogyakarta, Indonesia; 4 ValueCare BV, Utrecht, The Netherlands; 5 Department of radiation and oncology, The Netherlands Cancer Institute, Amsterdam, The Netherlands; 6 Department of radiotherapy, Dr Sardjito General Hospital/Faculty of Medicine, Universitas Gadjah Mada, Yogyakarta, Indonesia; 7 Department of oral and maxillofacial surgery, Academic Medical Centre, Amsterdam, the Netherlands; Dresden University of Technology, Germany

## Abstract

**Introduction:**

Nasopharyngeal carcinoma (NPC) has a high incidence in Indonesia. Previous study in Yogyakarta revealed a complete response of 29% and a median overall survival of less than 2 years. These poor treatment outcome are influenced by the long diagnose-to-treatment interval to radiotherapy (DTI) and the extended overall treatment time of radiotherapy (OTT). This study reveals insight why the OTT and DTI are prolonged.

**Method:**

All patients treated with curative intent radiotherapy for NPC between July 2011 until October 2012 were included. During radiotherapy a daily diary was kept, containing information on DTI, missed radiotherapy days, the reason for missing and length of OTT.

**Results:**

Sixty-eight patients were included. The median DTI was 106 days (95% CI: 98−170). Fifty-nine patients (87%) finished the treatment. The median OTT for radiotherapy was 57 days (95% CI: 57–65). The main reason for missing days was an inoperative radiotherapy machine (36%). Other reasons were patient’s poor condition (21%), public holidays (14%), adjustment of the radiation field (7%), power blackout (3%), inoperative treatment planning system (2%) and patient related reasons (9%). Patient’s insurance type was correlated to DTI in disadvantage for poor people.

**Conclusion:**

Yogyakarta has a lack of sufficient radiotherapy units which causes a delay of 3–4 months, besides the OTT is extended by 10–12 days. This influences treatment outcome to a great extend. The best solution would be creating sufficient radiotherapy units and better management in health care for poor patients. The growing economy in Indonesia will expectantly in time enable these solutions, but in the meantime solutions are needed. Solutions can consist of radiation outside office hours, better maintenance of the facilities and more effort from patient, doctor and nurse to finish treatment in time. These results are valuable when improving cancer care in low and middle income countries.

## Introduction

Cancer is the leading cause of death worldwide. About 70% of all cancer deaths occur in middle and low-income countries [1]. Although this burden increases every year, health-care systems in these countries are even at present not prepared for the high number of patients. In high-income countries survival rates are increasing due to improving treatment facilities and protocols, while low-income countries lack facilities and medication. Besides, funding for research on how to cope with these limitations is missing. The gap in treatment results between high-income and low-income countries is therefore widening [2].

While many politicians and scientists have emphasized the need to improve cancer care in low and middle-income countries, only a minority reported about the actual problems [3]–[5]. Before cancer care in these countries can be improved, research should focus on the current limitations in these health-care systems.

Indonesia is a low to middle-income country in which nasopharyngeal cancer (NPC) is one of the commonest types of cancer. Although NPC is highly sensitive to radiotherapy and chemo-radiotherapy, a previous study in Yogyakarta, Indonesia showed that the complete treatment response was less than 29% directly after therapy, compared to a 3-year-disease-free survival of 70% in international studies. Furthermore, the median overall survival in Yogyakarta was less than 2 years (21 months), compared to a 3-year overall survival of 80% in international literature [6]–[8].

Two important problems encountered in the studies in Yogyakarta were a diagnosis-to-treatment-interval of approximately 4 months and an overall radiotherapy treatment time of 62 days, which is 15–17 days too long. Optimally, a total dose of 66 to 70 Gray should be given in 33 to 35 fractions in a maximum 45 to 47 days. For each day by which radiotherapy treatment is extended, effective dose is lost, and the success rate declines rapidly [9]–[11].

As greater insight into the current problems in Indonesia’s cancer-care system might support the search for solutions, this study aims to explore reasons for the extended overall radiotherapy treatment time (OTT). Also the actual length of the OTT and the diagnose-to-treatment interval (DTI) are evaluated and factors that influenced these will be identified. In particularly, the association with type of insurance was investigated, since there is a wide variation in patient’s financial resources and these might have a great impact on DTI and OTT.

## Methods

### Patient Population

An official letter with confirmation of exemption of review was obtained from the medical ethical board of Dr. Sardjito Hospital, Universitas Gadjah Mada, Yogyakarta. All patients treated with curative intent radiotherapy for NPC in the period from July 2011 until October 2012 in Dr. Sardjito Hospital were included in this study. Sixty-eight patients were analyzed for inclusion. One patient never started radiotherapy and was excluded from all analysis. A total of 67 patients met the inclusion criteria. Information on patient characteristics was obtained by the medical chart. All data was analyzed anonymously. In table 1, patient characteristics and stage of disease are shown. Staging was performed by CT-scan of the head and neck, ultrasound of the abdomen, x-ray of the thorax and a bone scan. The median age was 47 years. Stage at diagnosis was available for 60 patients, advanced stage was predominantly seen. All patients were treated with 2D and/or 3D external beam radiotherapy in fractions of 2 Gray to a total dose 60–72 Gray.

**Table 1:** Patient and tumor characteristics.

Category	Subcategory	N = 67	
Sex	Male	47	70%
	Female	20	30%
Median age		47	IQR 40-60
Insurance type	Jamkesmasinsurance (poor)	15	22%
	Askes insurance(civil servants)	14	21%
	Self finance	38	57%
AJCC-stage	I	1	1%
	IIa	1	1%
	IIb	4	6%
	II	22	33%
	IVa	11	16%
	IVb	21	31%
	Missing	7	10%

IQR =  inter quartile range.

### Data Collection

The diagnosis-to-treatment interval (DTI) was calculated from the date a positive biopsy was obtained to the date radiotherapy was initiated. The overall treatment time (OTT) was calculated from time of radiotherapy initiation to the last day the patients received radiotherapy. During radiotherapy treatment, a nurse monitored daily the treatment of each patient and collected these data in a record form. This contained information on age, insurance type, the start date of radiotherapy and information on which days the patient did or did not get radiated. If the patient missed a treatment day, the reason for the missing day was recorded.

### Statistical Analysis

The major statistical endpoint of this study was the total number of radiotherapy days wherein all fractions of radiotherapy were administered. An assessment was made on the number of missed days and why this delay occurred. A comparison was made between patient and hospital related factors.

Secondary endpoint of this study was the length of the DTI. Both DTI and OTT were evaluated for correlation to insurance type. The insurance system in Indonesia is complex and differs per district. In general Yogyakarta has three types of insurances; jamkesmas (insurance for poor people), askes (insurance for civil servants) and patients with self-finance health care. Jamkesmas is a tax-funded health insurance, providing free health services in community health centres (puskesmas) and 3rd class (basic level) wards in government hospitals and some designated private hospitals. Expenses of health care will be paid if the head of the area confirms that the patient and the family have no resources and when the responsible doctor clarifies the need for health care. These approvals are time consuming and can cause a delay in diagnosis and treatment. Askes insurance is for civil servants, they contribute two per cent of their salaries and the government matches the contribution and covers almost all the diagnostic procedures and treatments [12]. Almost half of the Indonesian health care expenses are private health expenditures. This includes out of the pocket payment, private social insurance and other private insurance [13]. In this study we refer to these patients with the term ‘self-finance’.

To test for differences in distribution between the three insurance types the Kruskal Wallis test was used. When a difference was found the Mann-Whitney U was used to identify which group differed. For statistic analysis SPSS version 20 was used and a p-value of less than p = 0.05 was defined as significant.

## Results

### Patients

Sixty-eight patients were evaluated for inclusion in this study. One patient was excluded because she never started radiotherapy because of a poor physical condition, accordingly 67 patients were included. The median age was 47 years (range 9–81, inter quartile range (IQR) 40–60).

Type of insurance was distributed as followed; 38 patients (57%) financed their health care themselves, 15 patients (22%) had jamkesmas insurance and 14 patients (21%) had askes insurance.

Fifty-nine patients finished radiotherapy treatment. Eight patients did not finish treatment; of them 4 patients refused further treatment due to anxiety for side effects, 1 patient went to another hospital, 1 patient died after 12 fractions of radiotherapy and 2 patients never returned to the hospital for unknown reason.

One patient received 18 Gray from March to April 2011, but then stopped for unknown reasons and started treatment again in September 2011 and received another 70 Gray. In this case, for calculation of DTI the start date of the first treatment was used. For OTT and analysis of the missed days the second course was used.

### Diagnosis-to-treatment Interval

The precise date of biopsy proven diagnosis of NPC could not be retrieved in 3 patients. These patients were excluded for calculation of DTI. The median DTI was 106 days (IQR 53-66). Table 2 presents the median DTI and the start day of the radiotherapy treatment. Table 3 presents the day on which the radiotherapy was initiated.

**Table 2:** Diagnosis-to-treatment interval.

DTI in days	Median in days	Mean in days
Total (n = 64)	106 (IQR 38–176)	134 (95% CI: 98−170)
Jamkesmas insurance (poor)	192 (IQR 125–279)	214 (95% CI: 143−284)
Askes insurance (civil servants)	122 (IQR 51–143)	107 (95% CI: 72−143)
Self finance	54 (IQR 24–136)	111 (95% CI: 55−167)

DTI = diagnosis-to-treatment interval.

**Table 3:** Day of initiation of the radiotherapy treatment.

Start day (n = 67)	Number of patients (percentage)
Monday	26 (39%)
Tuesday	13 (19%)
Wednesday	15 (22%)
Thursday	12 (18%)
Friday	1 (2%)

**Figure 1:**
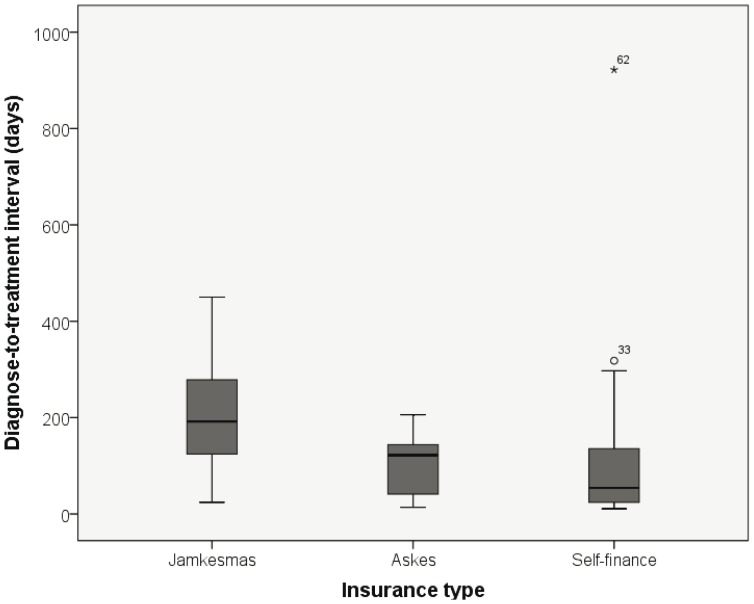
Distribution of diagnose-to-treatment interval and insurance type.

A difference in median and mean DTI was seen between the different types of insurance (table 2, figure 1). Kruskal Wallis test showed a significant difference between the three types of insurance (p = 0.003). Mann-Whitney U test showed that patient’s with jamkesmas insurance had a significant longer DTI than patients who had askes insurance (p = 0.016) and who financed their health care themselves (p = 0.001). Although a difference of 68 days between askes and self finance was seen, no significant difference was found (p = 0.325). One patient with private insurance had an interval to radiotherapy of 922 days (*62 in figure 1). She went first for alternative treatment with herbal medicine. Excluding this patient will make the differences between the insurance types stronger, although for askes versus self finance still no significance was achieved (p = 0.243).

### Overall Treatment Time

**Figure 2:**
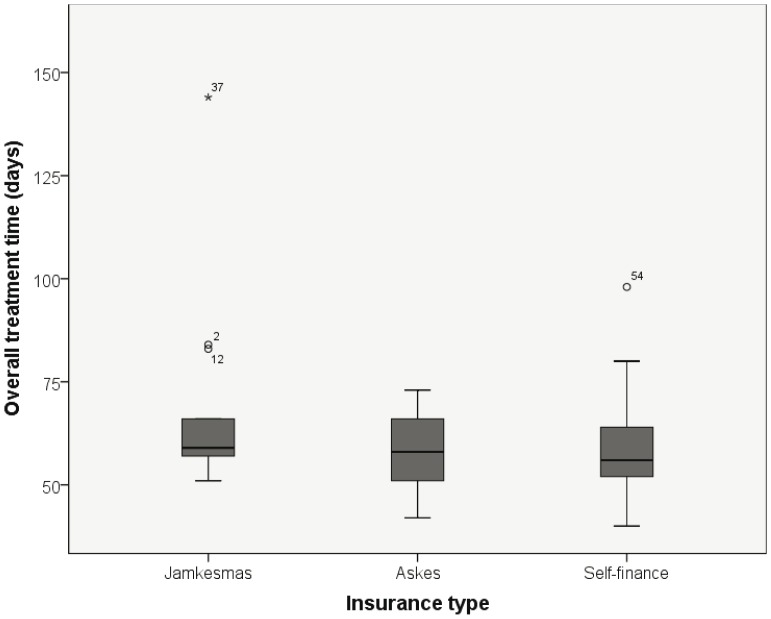
Distribution of overall treatment time and insurance type. The dark line in the middle of the boxes is the median. The bottom and top of the boxes indicate the 25th and the 75th percentile. The T-bars are the inner fences of all subjects and extend to a maximum of 1.5 times the inter quartile range. * are outliers until 3 times the inter quartile range. ^0^ are outliers exceeding 3 times the quartile range.

For 59 patients the OTT was analysed. The median OTT was 57 days. In total 584 days were missed, with a mean of 10 days per patient. A difference in median and mean OTT was seen between the different types of insurance, in favour of the patients with self finance (table 4, and figure 2). Kruskal Wallis test showed no significant difference (p = 0.28). One patient with jamkesmas insurance (*37 in figure 2) had an OTT of 144 days due to a poor physical condition. Excluding his patient did not change the significance of OTT versus insurance type.

**Table 4:** Overall treatment time and missed days.

OTT in days (n = 59)	Median in days	Mean in days
Total	57 (IQR 53−66)	61 (95% CI: 57–65)
Jamkesmas insurance (poor)	59 (IQR 57−66)	68 (95% CI: 53–83)
Askes insurance(civil servants)	58 (IQR 51−66)	58 (95% CI: 52–64)
Self finance	56 (IQR 52−64)	59 (95% CI: 55–63)
Number of missed daysper patient	8 (IQR 4−13)	10 (95% CI: 8–12)
Dose (in Gray)	66 (range 60–72)	67 (95% CI: 66-68)

OTT =  overall treatment time, IQR =  inter quartile range, CI =  confidence interval.

Table 5 presents the reasons for the missed days of treatment per category. An inoperative radiotherapy machine was the most frequent seen reason for missing treatment days. On 31 days the treatment planning system (TPS) was inoperative or there was a power black out. Forty-three patients (73%) missed one or more days because of problems with the facilities; an inoperative radiotherapy machine, TPS or a power black out.

**Table 5:** Reasons for missed treatment days.

Reason for missed days (n = 59)	Number of missed days	Percentage of totalmissed days	Number of patients	Percentage number of patients
Radiation system was inoperative	212	36.3%	40	67.8%
TPS was inoperative	11	1.9%	9	15.3%
Black out	20	3.4%	14	23.7%
Patient’s poor physical conditions	125	21.4%	23	39.0%
Calendar holiday	83	14.2%	39	66.1%
Adjustment of radiation field	40	6.8%	26	44.1%
Patient related	55	9.4%	13	22.0%
Other	10	1.7%	4	6.8%
Unknown	28	4.8%	3	5.1%

TPS =  treatment planning system.

The poor physical condition of the patient was the next most frequent reason for treatment extension, 23 patients (39%) were delayed because of their clinical condition. In all these cases the poor condition was related to therapy or treatment. The third most frequent reason was calendar holidays, 39 patients (66%) were delayed for this matter.

When 2-dimension radiotherapy is given the radiation field has to be adjusted after 40 Gray to protect the spinal cord, 26 patients (44%) missed one or more days because of these adjustments.

Patient related causes for a prolonged treatment were seen in 10,5%. Reasons were an extra day off after calendar holidays (n = 7, 17 days), to take care of their family (n = 2, 3 days), because of lack of financial resources to pay for the treatment or transport to the hospital (n = 4, 29 days) and 1 patient had a motor accident (n = 1, 6 days). Other reasons were miscommunications about the moment when facilities were in progress again (n = 2, 2 days), a shift from 2 dimensional radiation to 3 dimensional radiation (n = 1, 1 day) or problems with the chemotherapy (n = 2, 7 days).

## Discussion

This is the first study showing that the DTI and the OTT for radiotherapy in Indonesia are extended compared to international standards. This can be an explanation for the poor treatment outcome of NPC in Yogyakarta. At present, the answer to locally advanced NPC, which is one of the most common cancers in Indonesia, is concurrent chemo-radiation. Chemotherapy can be administered in most well equipped hospitals within Indonesia, whereas radiotherapy for most patients is limited. In several centres radiotherapy can be administered according to international standards, but the scarcity makes this only available for a very limited number of patients. In 2008 there were 25 units (accelerator and cobalt) available in Indonesia with a population of 229 million, whereas 6 were under commission, resulting in 0.13 units per million inhabitants [14]. This is in stark contrast with Europe, where in high-resource countries 5.5 accelerators are available per million inhabitants, 3.5 in medium-resource countries, and 2 per million in low resource countries [15]. The recommended number of treatment units per population differs widely; European guidelines recommend on average 5.9 units per million inhabitants [16], [17].

The vast lack of equipment, inadequate treatment procedures and preferential treatments for patients with self finance insurance results in excessive waiting lists. The very wealthy patients choose to receive their treatment in hospitals abroad, resulting in an outflow of substantial health care dollars to adjacent countries such as Malaysia, Singapore, and Australia [18]. Due to this loss in health care dollars, the less fortunate individuals cannot receive this quality of treatment. Currently, the Indonesian government offers a reimbursement for lower incomes, placing even more pressure on waiting lists [12].

In this study the median DTI was 3 to 6 months (95% CI for all patients). This is too long when comparing to international standards, with quality indicators of DTI of maximum 1 month [19]. Chen at al. showed in a systematic review that a delay in starting radiotherapy for head and neck cancer was significantly associated with higher recurrence rates and lower survival. They found an absolute increase in the risk of local recurrence of 3,7% per month delay [20]. The median intervals presented in those studies were all less than 2 months. With the intervals presented in our study and the high number of patients with already advanced disease at diagnosis, the risk on recurrence and poor survival is expectantly even higher, since the higher possibility of disease progression to an even more advanced stage [21], [22]. In a sequel study we would like to reveal the disease progression during the waiting time, since we expect that a number of patients might have progression to a incurable stage.

The length of DTI was associated with type of insurance. Poor patients with jamkesmas insurance had significantly longer delay to treatment, varying between 5 to 9 months (95% CI). This might be caused by the management of healthcare within jamkesmas insurance. After the positive biopsy for NPC, staging has to be performed by CT-scan of the head and neck region, ultra sound of the abdomen, bone scan and x-ray of the thorax. With jamkesmas insurance the government and treating doctor have to give approval for every diagnostic investigation and treatment. A solution to overcome this problem could be a diagnostic package deal, including all needed studies when biopsy is proven positive for NPC. This will shorten the DTI cause patients can be put on the waiting list for radiation earlier.

Another reason for the difference between the insurance types is that more resourced patients get a priority treatment. There is a different waiting list for radiotherapy for every type of insurance. Patients who can pay the treatment out of the pocket can even start within 2 weeks. Adaptations in the management for the poor will be beneficial in reducing the delay to complete diagnosis, but the absence of sufficient radiation units will still be a problem.

The OTT was 57 days, 10–12 days longer than recommended. Optimally, a total dose of 66 to 70 Gray should be given in 33 to 35 fractions and the best therapy response is achieved when the total dose is administered in 45 to 47 days. Each day of prolongation of the radiotherapy has a detrimental effect by a loss of the effective dose. During prolonged intervals tumour cells repopulate and will therefore influence the treatment success to a great extent [9], [10], [23]. Platek et al. presented in patients treated with radiation for head and neck cancer, an 8-fold increase on loco-regional progression if treatment time was prolonged to >57 days gave. Their results are in accordance to literature were the detrimental effect of treatment interruption on survival and local control vary between 1–5% per day [23].

The main reasons for the prolonged OTT were the radiotherapy facilities being intermittently operational (48% of the days were missed due to inoperative radiotherapy machines or treatment planning system, power black out and readjustment of radiation field). The hospital is already aware of the intermittently operating radiotherapy facility and got a maintenance contract for the radiation unit 4 months before start of this study. This contract is still going on. We see some improvement when comparing to earlier data of an OTT of 62 days, although better maintenance is still needed [6].

The next most frequent reason for an extended OTT was the patient’s physical condition due to the disease and side effects of the treatment. A next study has to investigate the actual physical problems, to find interventions to keep the patients fitter during treatment. By our experience we know that the nutritional status of some patients is poor and might worsen due to low intake by swallowing problems due to side effects of the treatment. To decrease the number of missed days due to patient’s condition, protocols to be more aggressive with earlier start of tube feeding and/or anti-fungal treatment of oral mucositis might give improvement.

Other causes for the delay in OTT were poor efficiency regarding calendar holidays. There were no schedule adaptations, like radiation in weekends or two times daily, to compensate for these days. Besides, half of the patients started on a Wednesday or later in the week. When 30-33 radiation days are needed, it is better to start on Monday or Tuesday, since the OTT will extend with two extra days by including an extra weekend in the schedule.

Patient-related delay accounted for only a small fraction of the delay, although still 22% of the patients missed days due to this reason (in total 55 days for 13 patients). Remarkable is that the OTT of 57 days is 5 days shorter than in the previous study conducted in Yogyakarta. This can be the result of the maintenance contract for the radiotherapy facilities as noted before or of a reduction in patient related delay, due to the committed attention of the monitoring nurse, who phoned the patient when they did not show up for treatment. With more awareness of the patients on the importance of a short OTT, the number of patient-related missed treatment days can be reduced further.

Differences between the types of insurance were found for OTT, although not significant (56, 58, 59 days, respectively self finance, askes and jamkesmas). This implies that all patients suffer in the same amount from the factors contributing to an extended OTT. There might be a significant difference when the study population is larger, since there is a trend in benefit for the self finance group. The reason for this benefit could be that these wealthier patients might have more counselling during treatment, accordingly physical deterioration will be noticed earlier and they have sufficient resources for supplemental nutrition and blood transfusions. Patient related reasons, like no money for transport will also be less among the richer patients.

With radiotherapy remaining the cornerstone of cancer treatment today, the results found here confirm that patient with NPC cannot be treated effectively. Probably, this also holds for the majority of other cancer patients, because most types of cancers need radiotherapy [24]. At the moment of initiation of radiotherapy the stage of disease might already be incurable. Full dose radiotherapy schedules for these patients have to be prevented. Future projects have to analyse if the current diagnostic system needs adjustments. Screening for distant metastasis should be repeated after the long waiting time, just before start of radiotherapy, since patients with distant metastasis are beyond cure and will be better served with adequate palliation. By only treating patients with a fair chance for cure the waist of limited resources can be prevented and shorten the waiting time.

The best way to address the current concerns in Indonesia should be to create sufficient radiotherapy facilities with well-trained staff, and raise awareness on the effect of an extended DTI and OTT. The growing economy in Indonesia will expectantly in time, enable these solutions, although it will take at least a decade to accomplish this, since also bunkers has to be build and doctors, nurses and technical staff have to be trained. In the meantime small-scale and easy to implement solutions are needed. Possible solutions could be more radiation hours per treatment unit outside office hours, in weekends and on holidays, better maintenance of the treatment units and planning system, including a power back up system and more awareness and dedication of the doctor, patient and nurse to complete the treatment in 47 days. We think that the problems found in this study are not specific for the treatment of nasopharyngeal cancer in Yogyakarta, Indonesia only. Also when treating other types of cancer which need radiation treatment or in other regions with comparable health systems, the same problems might be encountered. Therefore, these results can be helpful when facing the challenges in improving the treatment of cancer in other low- and middle income countries.
